# γ-GC mitigates MASLD by reducing CD36 palmitoylation and plasma membrane translocation mediated by AKT/KLF10/zDHHC7 axis

**DOI:** 10.1186/s12986-026-01173-6

**Published:** 2026-07-12

**Authors:** Jinyi Zhou, Chen Yang, Yangyu Guo, Lishuang Zhao, Shuai Lu, Lan Luo, Da Zhou, Zhimin Yin

**Affiliations:** 1 Central Laboratory, The Second Affiliated Hospital of Nanjing, University of Chinese Medicine, Nanjing, China; 2 College of Life Science, Jiangsu Province Key Laboratory for Molecular and Medical Biotechnology, Nanjing Normal University, No.1 Wenyuan Road, Nanjing, 210046 People’s Republic of China; 3 Department of Gastroenterology and Hepatology, Zhongshan Hospital, Fudan University, Shanghai, 200032 People’s Republic of China; 4 State Key Laboratory of Pharmaceutical Biotechnology, School of Life Sciences, Nanjing University, Nanjing, 210023 China; 5 Department of Biology, School of Basic Medical Science, Guizhou Medical University, Guiyang, 550000 People’s Republic of China

**Keywords:** γ-GC, MASLD, Hepatic steatosis, CD36, Posttranslational modification

## Abstract

**Background:**

Metabolic dysfunction-associated steatotic liver disease (MASLD) is a worldwide disease characterized by hepatic steatosis. γ-glutamylcysteine (γ-GC), a non-protein thiol peptide with nontoxic side effects, can alleviate insulin resistance (IR) and alcoholic liver disease (ALD). However, the role of γ-GC in MASLD remains elusive. Herein, the aim of this study was to reveal the effects and underlying molecular mechanisms of γ-GC in the progression of MASLD.

**Methods:**

In vivo model of mice fed with high-fat diet (HFD) and in vitro model of hepatocytes treated with FFA (oleic acid/palmitic acid = 2/1) were established to examine the therapeutic effects and molecular mechanisms of γ-GC in MASLD.

**Results:**

HFD mice develop dyslipidemia and hepatic steatosis due to obesity. Increased lipogenesis-related protein expression and aggravated fatty acid uptake are also observed in hepatocytes treated with FFA. Mechanistic studies suggest that γ-GC can alleviate hepatic lipid deposition and steatosis through balancing lipid metabolism. Importantly, γ-GC inhibits KLF10-regulated zDHHC7 transcription via activating AKT signaling, thereby suppressing zDHHC7-mediated CD36 palmitoylation and plasma membrane translocation. Ultimately, γ-GC reduces CD36-mediated fatty acid uptake and attenuates FFA-associated lipogenic signaling, resulting in the amelioration of MASLD.

**Conclusions:**

In summary, our results support γ-GC as a candidate dipeptide for the treatment of MASLD, which alleviates hepatic lipid deposition and steatosis by inhibiting KLF10/zDHHC7-mediated CD36 palmitoylation and plasma membrane translocation through activation of AKT signaling.

**Supplementary Information:**

The online version contains supplementary material available at https://doi.org/10.1186/s12986-026-01173-6.

## Introduction

Nonalcoholic fatty liver disease (NAFLD) refers to a series of diseases with excessive lipid deposition in the liver, often accompanied by metabolic syndromes such as obesity, hypertension, hyperlipidemia and diabetes [1]. The prevalence of NAFLD is increasing globally due to the obesity epidemic and is expected to become the main reason for liver transplantation by 2030, while the cost of the healthcare system is also increasing [2]. As the research on the pathogenesis of NAFLD deepens, the “multi-hit” theory has been gradually accepted. In fact, numerous factors, including genetics, air pollution, dietary habits, alcohol consumption and IR, have been identified as contributors to the development of NAFLD [3, 4]. In 2023, NAFLD was officially renamed MASLD [5]. Lipid deposition, a main characteristic of MASLD, is primarily caused by excessive free fatty acids (FFAs) being transported to the liver and stored there in the form of triglyceride (TG) [6]. Therefore, elucidating the source of FFAs in the liver is essential for future treatment of MASLD.

The sources of hepatic FFAs mainly include fatty acid uptake, de novo lipogenesis (DNL), and diet. Approximately 60% of FFAs, that converted into TG in the liver come from fatty acid uptake. In addition, increasing evidence has also emphasized the important role of hepatic DNL in the development of MASLD; however, the mechanism regulating hepatic DNL are not fully understood [7]. Fatty acid translocase (FAT/CD36), a multifunctional receptor located on the plasma membrane, has been reported to be involved in the pathophysiology of the heart and liver and plays an important role in fatty acid uptake and DNL [8, 9]. As CD36 is highly expressed in MASLD patients and is proportional to the degree of hepatic steatosis, it has been shown to play an important role in hepatic steatosis and is closely related to the palmitoylation of its C and N terminals [10]. Protein palmitoylation is a reversible posttranslational modification of lipids usually controlled by a member of the Asp-His-His-Cys (DHHC) family of protein palmitoyl acyl transferases (PATs) [11]. Dynamic palmitoylation of CD36 has been reported to regulate the process of cellular fatty acid uptake via mediating membrane endocytosis [12]. In addition, palmitoylation of CD36 increases its translocation from cytoplasm to plasma membrane, suggesting that reducing the palmitoylation of CD36 can ameliorate MASLD through alleviating the intracellular lipid deposition and inflammatory cascade to inhibit lipid metabolic disorders [13].

Glutathione (GSH), an essential non-enzymatic antioxidant in mammalian cells, can act as a cofactor for antioxidant and detoxification enzymes to protect cells against free radicals and pro-oxidants. In addition, GSH has been reported to be involved in other cellular processes such as protein folding, degradation of proteins with disulfide bonds, cell cycle regulation, apoptosis and ferroptosis [14]. Yeast is rich in GSH. In addition, some fruits and vegetables rich in vitamin C, resveratrol and flavonoids also have antioxidant capacity, which can promote the synthesis of GSH [15–17]. As GSH can be broken down into a single amino acid in the gut, exogenous GSH administration does not increase intracellular GSH levels; however, γ-GC, as the direct synthetic precursor of GSH, can be readily taken up by many cell types and catalyzed into GSH by glutathione synthetase (GSS) without being affected by γ-glutamylcysteine synthetase [18]. In addition to being converted into GSH, γ-GC itself also has a powerful antioxidant effect and plays an important role in inflammation, aging, diabetes and other diseases. Therefore, γ-GC has the same potential advantages as GSH in preventing or treating some related diseases [19–22]. Recently, an increasing number of studies have found that γ-GC also participates in hepatoprotection, such as reducing metal-induced hepatocyte damage and protecting ALD induced by ethanol [23]; however, whether γ-GC has the potential to mitigate MASLD remains unclear.

Herein, γ-GC was found to have a protective effect on mice with MASLD, and was able to ameliorate lipid ectopic deposition, hepatic steatosis and other metabolic parameters induced by HFD in mice. Subsequently, we explored the molecular mechanisms of γ-GC involved in alleviating MASLD both in vivo and in vitro. Our results demonstrated that γ-GC inhibits KLF10/zDHHC7-mediated CD36 palmitoylation and plasma membrane translocation via AKT activation, thereby blocking CD36-dependent fatty acid uptake and attenuating lipogenesis-related signaling, ultimately alleviating obesity-induced MASLD.

## Materials and methods

### Animal models

The research followed the ARRIVE guidelines, and the Animal Ethics Committee of Nanjing Normal University approved all animal experiments [IACUC-20230701]. Mice were monitored routinely for general health and signs of distress (posture, coat condition, activity, respiration, and stool consistency). Humane endpoints were predefined (e.g., > 15–20% body-weight loss, persistent lethargy, severe dehydration, or inability to access food/water), and any animal meeting these criteria would be humanely euthanized.

γ-GC used in this study was enzymatically synthesized from monosodium L-glutamate and L-cysteine in the presence of ATP, using γ-glutamylcysteine synthetase as the catalyst, as previously described [22]. The final product was obtained at a yield of 36 g/L, with a purity of 79% as determined by HPLC.

The animal experiments were essentially carried out as described in detail previously [24]. 5-week-old C57BL/6J male mice, weighing 18–22 g, purchased from GemPharmatech Co., Ltd (Nanjing, China), were raised in an SPF environment with free eating and drinking. After adaptive feeding for 1 week, all mice were randomly divided into five groups (*n* = 7–8 mice/group): NCD + vehicle, NCD + γ-GC (H), HFD + vehicle, HFD + γ-GC (L), and HFD + γ-GC (H). Mice in the HFD groups were fed a 60 kcal% fat diet. γ-GC was administered once daily by oral gavage at 250 mg/kg (L) or 500 mg/kg (H). Vehicle groups received 0.9% saline by oral gavage at an equivalent volume. The gavage was performed once daily throughout the 16-week feeding period at approximately the same time each day by trained personnel to minimize procedure-related variability and stress. After 16 weeks, mice were fasted 12 h in advance and then anesthetized with sodium pentobarbital (30 mg/kg). Following deep anesthesia, blood was collected from the retro-orbital sinus. The liver and other tissues were weighed and stored in 4% paraformaldehyde (PFA) or frozen directly at -80 °C for further assays.

### Antibodies, chemicals and plasmids

Antibodies against zDHHC4 (1:1000, A17980), zDHHC5 (1:1000, A18114), zDHHC7 (1:1000, A17981), p-AKT (Ser473) (1:1000, AP0637), AKT (1:1000, A11016), Na^+^/K^+^-ATPase (1:1000, A11683), β-actin (1:10000, AC026), HRP Goat Anti-Rabbit IgG (1:5000, AS014) and HRP Goat Anti-Mouse IgG (1:5000, AS003) were purchased from Abclonal (Wuhan, Hubei, China). Antibodies against CD36 (1:1000, BS90237) was purchased from Bioworld Technology (Minneapolis, MN, USA). Antibodies against KLF10 (1:1000, CY8099) was purchased from Abways (Shanghai, China). Antibodies against ACC1 (1:1000, #3676) and FASN (1:1000, #3180) were purchased from Cell Signaling Technology (Beverly, MA, USA). Antibodies against PPARγ (1:1000, 16643-1-AP), SREBP-1c (1:1000, 14088-1-AP) and LXRα (1:1000, 14351-1-AP) were purchased from Proteintech (Chicago, IL, USA).

Palmitoylation inhibitor 2-bromohexadecanoic acid (2-BP, T35364) and AKT inhibitor MK-2206 monohydrochloride (MK2206, T62609) were purchased from Topscience (Shanghai, China). 60 kcal% fat diet was purchased from Research Diets (New Brunswick, NJ, USA). Hanks’ Balanced Salt Solution was purchased from Beyotime (Beijing, China). N-[6-(biotinamido) hexyl]-3’-(2’-pyridyldithio) propionamide (Biotin-HPDP), type Ⅳ Collagenase and type Ⅰ Collagen were purchased from Yuanye (Shanghai, China). N-ethylmaleimide (NEM) and hydroxylamine (50% aqueous solution) were purchased from Macklin (Shanghai, China). Oil red O (ORO), oleic acid (OA) and palmitic acid (PA) were purchased from Sigma-Aldrich (St. Louis, MO, USA). TG, total cholesterol (TC), NEFA, high-density lipoprotein cholesterol (HDL-C), low density lipoprotein cholesterol (LDL-C), alanine aminotransferase (ALT/GPT) and aspartate aminotransferase (AST/GOT) assay kits were purchased from Jiancheng Bioengineering Institute (Nanjing, Jiangsu, China).

The pGL3-Basic-hzDHHC7 promoter was purchased from the Public Protein/Plasmid Library laboratory (Nanjing, Jiangsu, China).

### Histology and immunohistochemistry

According to the previous method [22], paraffin-embedded liver and adipose tissue samples were sectioned and stained by using H&E for histological examination. Hepatic steatosis was graded on H&E-stained sections according to the NAS-based system on a 0–3 scale (grade 0: <5%; grade 1: 5–33%; grade 2: 34–66%; grade 3: 67–100% of hepatocytes affected). For each tissue, 3 non-overlapping fields were randomly selected from 10 sections per liver lobe at ×200 magnification (avoiding large vessels and bile ducts). Steatosis scores were independently assessed by two blinded observers. Discrepancies were resolved by consensus (or the mean of two scores was used for analysis) [25].

For immunohistochemistry, liver tissue sections underwent deparaffinization and rehydration, followed by antigen retrieval and inhibition of endogenous peroxidase activity using 3% hydrogen peroxide. After blocking nonspecific binding sites, the slides were incubated with the primary antibody at 4 °C overnight. Subsequently, they were treated with a biotinylated secondary antibody and a third antibody conjugated with SABC (BosterTech, China). The sections were then developed using a DAB staining kit and counterstained with hematoxylin. Finally, the stained sections were visualized and captured under a light microscope (Nikon, Japan).

### Bioinformatics analysis

We performed a Python-based reanalysis of the public human liver transcriptome cohort GSE48452 and GSE89632, which includes histologically defined groups healthy controls (Control), simple steatosis (Steatosis), and NASH (MASH). Processed expression values were used to evaluate differential expression of CD36, KLF10, and ZDHHC7 between Steatosis vs. Control and MASH vs. Control. Group contrasts were tested using t-statistics, and *P* values were adjusted for multiple testing using the FDR. To evaluate coordinated expression, we calculated Spearman rank correlations among CD36/KLF10/ZDHHC7 within Steatosis samples and within MASH samples separately, with BH adjustment across gene pairs.

### Cell culture and treatment

The isolation and culture of primary mouse hepatocyte were essentially carried out as described in detail previously [26]. Human hepatoma cell line HepG2 cells were maintained in EMEM (Wisent, Canada) with the addition of 10% (w/v) FBS (Wisent, Canada), 100 U/mL penicillin, 100 µg/mL streptomycin (Wisent, Canada). Cells were maintained at 37 °C with 5% CO_2_.

For FFA loading, OA and PA were prepared by conjugating them with fatty-acid-free BSA at a 2:1 molar ratio [27, 28]. Briefly, PA was dissolved in 0.1 M NaOH at 70 °C, and OA was dissolved in distilled water at 55 °C. The dissolved fatty acids were then added dropwise to a pre-warmed (55 °C) 10% (w/v) fatty-acid-free BSA solution in serum-free medium and stirred for 1–2 h until a clear stock solution was obtained. The final working concentrations of OA and PA in the culture medium were 667 µM and 333 µM (total 1 mM), respectively. Control cells were treated with a corresponding concentration of BSA vehicle. Cells were subjected to serum starvation by replacing the culture medium with FBS-free medium for 6 h prior to FFA stimulation; during serum starvation, the medium contained 0.5% fatty-acid-free BSA as indicated. Cells were exposed to 1 mM FFA for 24 h, followed by treatment with different doses of γ-GC for an additional 24 h.

### Cell viability

Cell viability/metabolic activity was assessed using the CCK-8 assay (Beyotime, China). Following treatment, CCK-8 reagent was added to each well and incubated. Absorbance was then measured using a microplate reader. To ensure reproducibility, the experiment was repeated three times independently.

### LDH release assay

Cytotoxicity was evaluated by measuring LDH release using an LDH cytotoxicity detection kit (Beyotime, China) according to the manufacturer’s instructions. Following treatment, culture supernatants were collected and incubated with the LDH reaction mixture. Absorbance was then measured using a microplate reader. To ensure reproducibility, the experiment was repeated three times independently.

### Measurement of GSH/GSSG ratio

Hepatic GSH/GSSG ratio (reduced glutathione/oxidized glutathione) was determined using frozen liver tissues. After euthanasia, liver tissues were rapidly excised, rinsed with ice-cold PBS to remove residual blood, blotted dry, divided into aliquots, snap-frozen in liquid nitrogen, and stored at -80 °C until analysis. Repeated freeze-thaw cycles during storage were avoided. For analysis, liver samples were processed on ice. Briefly, frozen liver tissues were weighed and homogenized in the protein removal reagent or assay buffer supplied with the GSH and GSSG assay kit according to the manufacturer’s instructions (Beyotime, Shanghai, China). The homogenates were lysed using the freeze-thaw procedure recommended by the manufacturer and then centrifuged at 12,000 × g for 10 min at 4 °C. The supernatants were collected for the determination of total glutathione and GSSG. Specific detection reagents were added to each sample according to the kit protocol, and absorbance was measured at 412 nm using a microplate reader. Total glutathione was expressed as GSH equivalents. Reduced GSH levels and the GSH/GSSG ratio were calculated according to the manufacturer’s instructions. The results were normalized to tissue weight or total protein content.

### Measurement of MDA content

Hepatic malondialdehyde (MDA) levels were also measured using frozen liver tissues prepared under the same collection and storage conditions described above. For MDA measurement, frozen liver samples were thawed and processed on ice. Liver tissues were homogenized in ice-cold PBS, and the homogenates were centrifuged at 12,000 × g for 10 min at 4 °C. The supernatants were collected for analysis using a lipid peroxidation MDA assay kit according to the manufacturer’s protocol (Beyotime, Shanghai, China). Absorbance was measured at 532 nm using a microplate reader. Protein concentrations were determined using a BCA protein assay kit, and hepatic MDA levels were normalized to total protein content.

### Immunoblotting

Immunoblotting analysis was performed following standard protocols [22]. Proteins were extracted from HepG2 cells and mouse liver using a lysis buffer (Beyotime, China) supplemented with protease and phosphatase inhibitors. After centrifugation at 12,000 × g for 15 min at 4 °C, the supernatants were collected and protein concentrations were determined using a BCA protein assay kit (Leagene, China). Equal amounts of protein (20–40 µg) were separated by 8%-12% SDS-PAGE and then transferred onto PVDF membranes. The membranes were blocked with 5% non-fat milk in TBST for 1 h at room temperature and then incubated overnight at 4 °C with the indicated primary antibodies. After washing with TBST, the membranes were incubated with HRP-conjugated secondary antibodies for 1 h at room temperature. Protein bands were visualized using an enhanced chemiluminescence reagent and captured using a chemiluminescence imaging system.

### ORO staining

Liver samples were preserved in 4% PFA and embedded in freezing medium (Leica). Section  (8 μm) and treated hepatocytes were stained with ORO solution (Sigma-Aldrich) for 20–30 min at room temperature, followed by rinsing with 60% isopropanol to eliminate excess dye. Hematoxylin was used for counterstaining, and images were acquired using a light microscope (Nikon, Japan). Quantification was performed using Image J. For each tissue, 3 randomly selected non-overlapping fields at ×100 magnification was analyzed under identical acquisition settings.

### Immunoprecipitation and acyl-biotin exchange (IP-ABE)

The IP-ABE assay was utilized to detect the palmitoylation of CD36, following a standard protocol [29]. Total proteins were extracted from cells and treated with NEM at 4 °C for 1 h with continuous agitation. The target protein was immunoprecipitated using a specific antibody and magnetic beads, then incubated with 1 M HAM (pH 7.2) at room temperature for 1 h. Subsequently, the protein was labeled with 5 µM biotin-BMCC at 4 °C for 2 h. The protein-bead complex was resuspended in 40 µL SDS sample buffer, boiled at 100 °C for 10 min, and analyzed via immunoblotting assay.

### Biochemical analysis

Serum levels of insulin, ALT, AST, HDL-C, LDL-C, TG and TC, were detected using commercial assay kits according to the manufacturer’s instructions. Serum glucose was analyzed using commercially glucose test strips (Sinocare, China). HOMA-IR was calculated using fasting glucose and fasting insulin.

TG, TC and FFAs contents in mouse liver and hepatocytes were also measured using commercial kits according to the manufacturer’s protocol.

### RNA extraction and real‑time PCR

Total RNA was isolated from cells and liver tissues with the RNA isolation kit (Takara, Shiga, Japan), and cDNA synthesis was performed using a reverse transcription kit (Vazyme Biotech, Nanjing, China) following the manufacturer’s protocol. Quantitative PCR (qPCR) was conducted on the ABI StepOnePlus Real-Time PCR system (Applied Biosystems, USA) with SYBR Premix Ex Taq (Vazyme Biotech, Nanjing, China). GAPDH served as the internal control. The primers for target genes were designed as follows:

Human *Zdhhc7*, sense: CCCAAAGGAAACGCTACGAAA,

Anti-sense: CGCGCTCGGGTTTAATACAG;

Human *Zdhhc4*, sense: TCTTCATGCTGGGCTTTGT,

Anti-sense: CACTCGTTAGTAGTCTGGTTGG;

Human *Zdhhc5*, sense: TTCTTGTCTGTTCTGCCGCT,

Anti-sense: CGGGACATACTTGCTGGGTT;

Human *Gapdh*, sense: ACCACAGTCCATGCCATCAC,

Anti-sense: TCCACCACCCTGTTGCTGTA.

### Statistical analysis

To ensure the reliability and consistency of the results, all experiments were performed using three independent biological replicates. Statistical differences between groups were evaluated using Student’s t-test and one-way ANOVA, followed by Tukey multiple comparisons test. Data are presented as mean ± SD, with *P* values indicating statistical significance (*P* < 0.05). GraphPad Prism 8.0 was utilized for all statistical analyses.

## Results

### γ-GC improves dyslipidemia in HFD mice

**Fig. 1:**
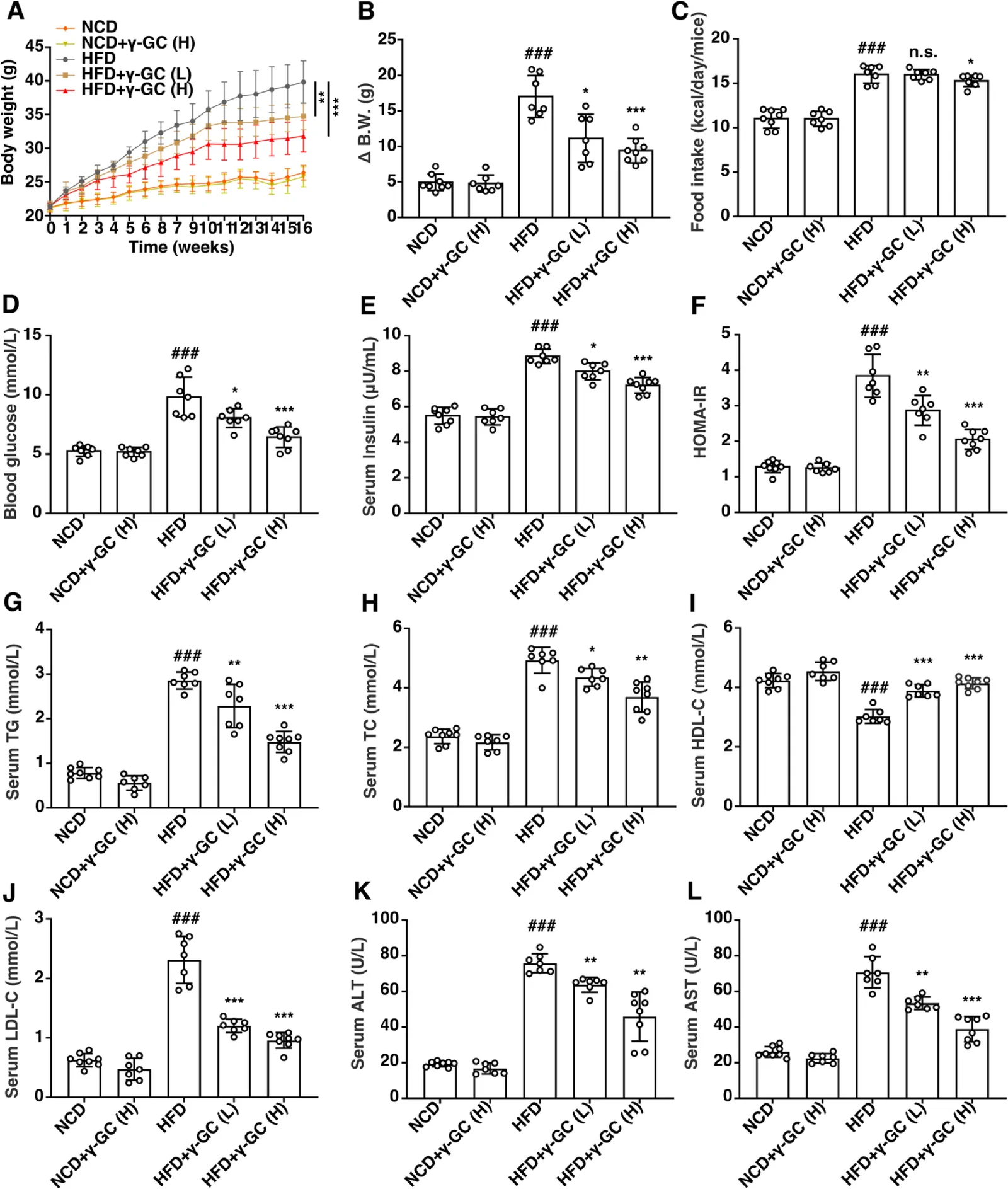
γ-GC improves dyslipidemia in HFD mice.Male C57BL/6J mice (5 weeks) were randomly divided into five groups: NCD group, NCD + γ-GC (H) group, HFD group, HFD + γ-GC (L) group, HFD + γ-GC (H) group. The body weight change curve of each mouse for 16 weeks (**A**). The body weight gain of each mouse after 16 weeks (**B**). Caloric intake of each mouse after 16 weeks (**C**). Fasting blood glucose level of each mouse at the 15th week (**D**). Serum insulin level of each mouse after 16 weeks (**E**). The index of HOMA-IR (**F**). Serum TG level of each mouse after 16 weeks (**G**). Serum TC level of each mouse after 16 weeks (**H**). Serum HDL-C level of each mouse after 16 weeks (**I**). Serum LDL-C level of each mouse after 16 weeks (**J**). Serum ALT level of each mouse after 16 weeks (**K**). Serum AST level of each mouse after 16 weeks (**L**). In (**A**-**L**), unpaired student’s t test was used for statistical analysis. Data are expressed as mean ± SD. ###*P* < 0.001 (vs. NCD group), *P < 0.05, **P < 0.01, ***P < 0.001 (vs. HFD group), n.s. not significant (*P* > 0.05)

To determine the effect of γ-GC on MASLD, we performed metabolic parameters analysis on C57BL/6J mice (fed a HFD or NCD for 16 weeks). Firstly, the body weight of each mouse was continuously monitored for 16 weeks, as Fig. 1A, B showed that γ-GC significantly inhibited body weight gain in obese mice induced by HFD. Further analysis showed that HFD mice treated with γ-GC also had significantly reduced food intake (Fig. 1C). IR is closely related to MASLD, and high IR was considered to be the most significant predictor of MASLD [30], and studies have showed that serum insulin level is closely related to hepatic balloon and hepatic lobular inflammation [31]. Therefore, we also measured the fasting blood glucose and the serum insulin level of each mouse. Results showed that γ-GC remarkably reduced fasting blood glucose (Fig. 1D) and serum insulin level (Fig. 1E) of HFD mice, especially high-dose γ-GC. At the same time, our investigation showed that γ-GC significantly reduced HOMA-IR value compared with HFD group, suggesting an improvement in fasting insulin resistance indices in HFD mice (Fig. 1F). After 16 weeks, each of mouse was euthanized, and some serum lipid indexes, including TG, TC, HDL-C, LDL-C levels were measured. We found that the serum levels of TG, TC and LDL-C in HFD mice were increased, accompanied by the decrease of HDL-C levels; while γ-GC treatment enormously reversed those phenomena (Fig. 1G-J). Further detection of serum ALT and AST levels, key indicators of liver function, showed that γ-GC significantly reduced the increase of serum ALT and AST levels caused by obesity in HFD mice, which suggesting that γ-GC has a potential liver protection effect (Fig. 1K, L). These above results indicated that γ-GC can inhibit dyslipidemia caused by HFD.

### γ-GC ameliorates HFD-induced obesity and hepatic steatosis

**Fig. 2:**
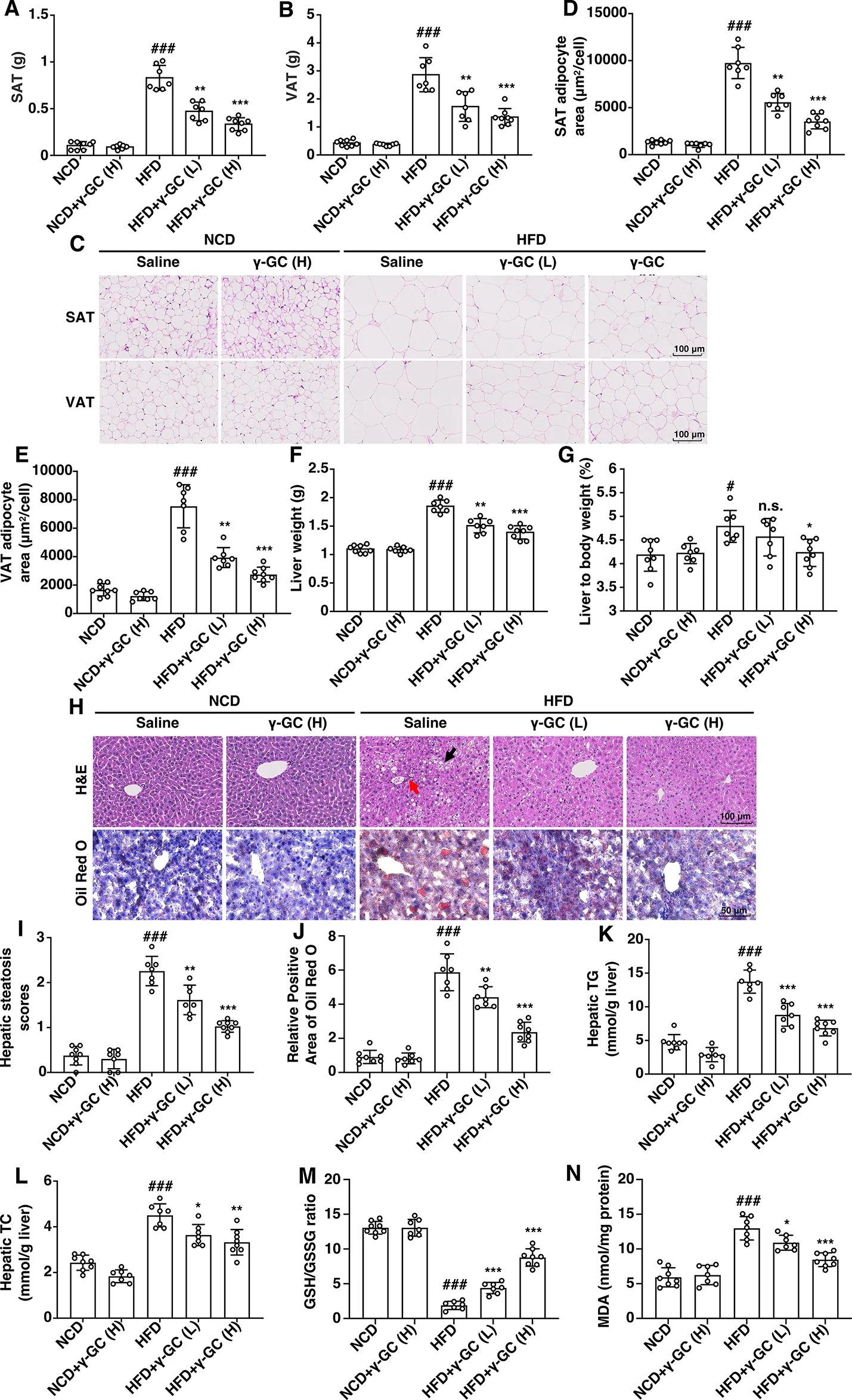
γ-GC ameliorates HFD-induced obesity and hepatic steatosis.The weight of SAT (**A**) and VAT (**B**). H&E staining of SAT and VAT (× 400). Scale bar: 100 μm (**C**). Adipocyte size in SAT (**D**) and VAT (**E**). Liver weight of each mouse (**F**). Liver coefficient: the percentage of liver weight to mice body weight (**G**). H&E staining of liver (× 400, Scale bar: 100 μm) and ORO staining of liver (Scale bar: 50 μm) (**H**). Hepatic steatosis scores based on the evaluation of H&E staining of mice liver, divided into 0–3 grades (grade 0: < 5%; grade 1: 5%-33%; grade 2: 34%-66%; grade 3: 67%-100%) (**I**). Quantitative analysis of Oil Red O staining (**J**). TG level in liver tissue of each mouse (**K**). TC level in liver tissue of each mouse (**L**). Total glutathione and reduced glutathione were quantified using the GSH assay kit (**M**). MDA levels were measured using a lipid peroxidation MDA assay kit (**N**). In (**A**, **B**, **D**, **E**, **F**, **G**, **I**, **J**, **K**, **L**, **M**, **N**), unpaired student’s t test was used for statistical analysis. Data are expressed as mean ± SD. #*P* < 0.05, ###*P* < 0.001 (vs. NCD group), *P < 0.05, **P < 0.01, ***P < 0.001 (vs. HFD group), n.s. not significant (*P* > 0.05)

Obesity resulting from a long-term HFD is a fatal factor that increases the risk of MASLD. Excessive accumulation of TG in adipose cells leads to the growth of subcutaneous adipose tissue (SAT) and visceral adipose tissue (VAT) [32]. To assess the effect of γ-GC on lipid deposition, we measured the weight of SAT and VAT. As shown in Fig. 2A and B, γ-GC treatment remarkably reduced the weight of SAT and VAT in HFD mice. Besides, H&E staining results also showed that the average size of adipose cells in HFD mice decreased significantly after γ-GC administration (Fig. 2C). It was also apparent that the adipocyte areas of SAT (Fig. 2D) and VAT (Fig. 2E) in γ-GC-treated HFD mice were both reduced to about 3 times than those in HFD mice. These results suggested that γ-GC can significantly inhibit the occurrence of obesity in HFD mice. As the accumulation of excess TG in hepatocytes is a key pathological feature of MASLD [33], the state of lipid accumulation in hepatocytes was also investigated. HFD-induced increases in liver weight (Fig. 2F), liver weight-to-body weight ratio (Fig. 2G) and lipid content (TG and TC) (Fig. 2K, L) were conceivably attenuated by γ-GC treatment. By observing the H&E-stained pathological sections of liver tissue and blindly grading the degree of steatosis, it was found that the number of fat vacuole (Fig. 2H) and hepatic steatosis score (Fig. 2I) of γ-GC-treated HFD mice were reduced, indicating that the degree of hepatic steatosis was reduced after γ-GC administration. ORO makes the lipid droplets red, which can intuitively observe the lipid deposition in tissue. To strengthen the histological assessment, we additionally quantified hepatic lipid deposition by ORO staining. Consistent with the H&E-based steatosis grades, the ORO-positive area was significantly increased in HFD mice and markedly reduced by γ-GC (Fig. 2H, J). Excess lipid accumulation in hepatocytes disrupts lipid metabolic homeostasis and promotes lipid peroxidation and oxidative stress. To verify the protective effect of γ-GC against hepatic oxidative stress, hepatic redox status was further assessed. As described in Fig. 2M and N, the GSH/GSSG ratio had a significant decrease, whereas the MDA level exhibited a remarkable increase in the HFD group compared with the NCD group. Interestingly, γ-GC treatment dramatically led to an elevation of hepatic GSH/GSSG ratio, as well as the reduction of hepatic MDA level compared to those in HFD group. The hepatic GSH/GSSG ratio was negatively correlated with hepatic steatosis score and MDA content, suggesting a link between redox restoration and metabolic improvement. Collectively, these results revealed that γ-GC administration dramatically ameliorates HFD-induced obesity and hepatic steatosis.

### γ-GC inhibits lipid deposition through regulating FFA-associated lipogenic signaling

**Fig. 3:**
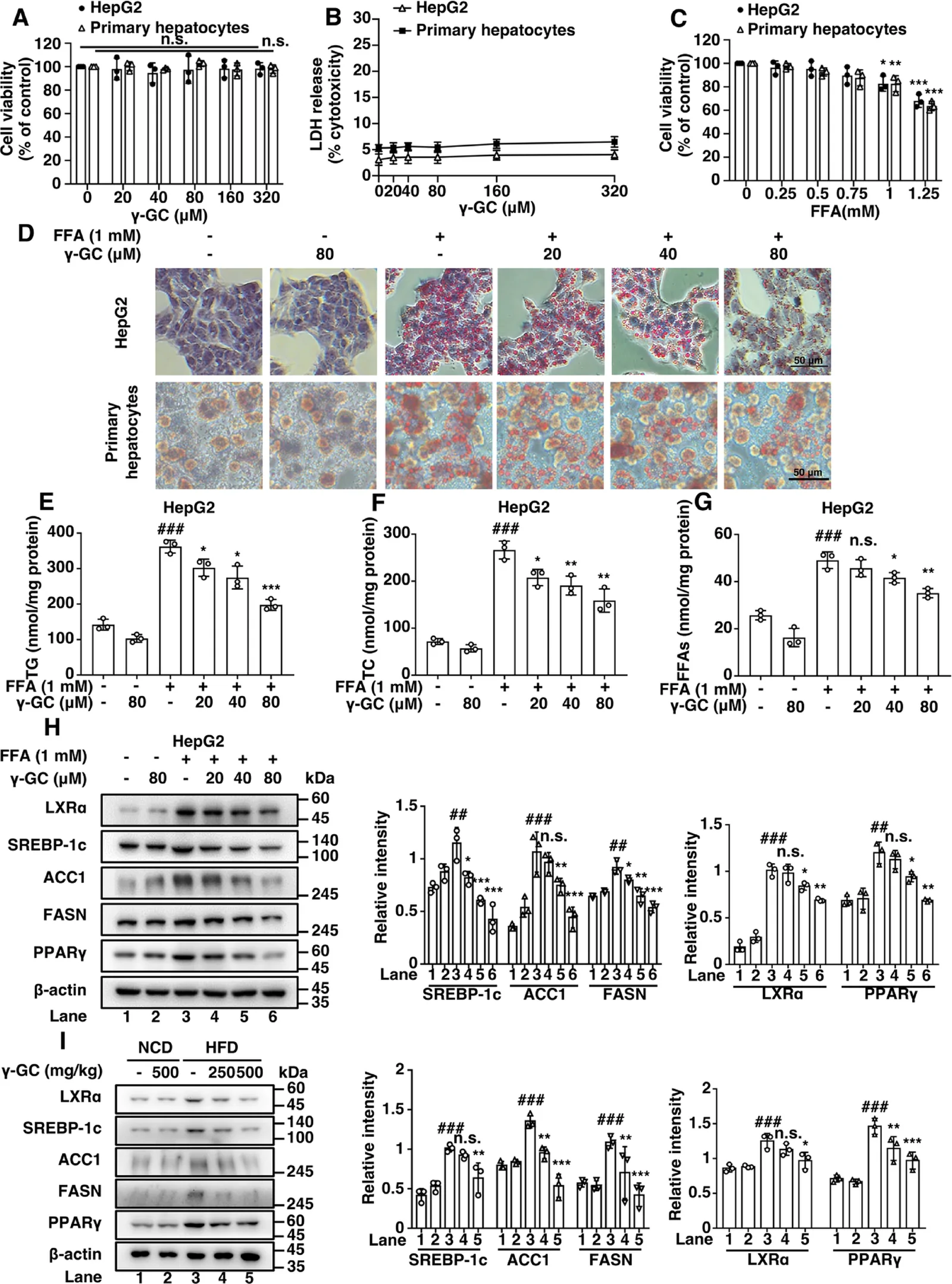
γ-GC inhibits lipid deposition through regulating FFA-associated lipogenic signaling.HepG2 cells and primary hepatocytes were stimulated with γ-GC for 48 h, CCK-8 assay was performed to test cell viability (**A**). LDH release assay was performed to detect membrane integrity-associated cytotoxicity (**B**). HepG2 cells and primary hepatocytes were stimulated with FFA for 24 h, CCK-8 assay was performed to test cell viability (**C**). HepG2 cells or primary hepatocytes were stimulated with 1 mM FFA for 24 h, then treated cells with different doses of γ-GC (0, 20, 40, 80 µM) for another 24 h. ORO staining of HepG2 cells and primary hepatocytes (Scale bar: 50 μm) (**D**). TG level in HepG2 cells (**E**). TC level in HepG2 cells (**F**). FFAs level in HepG2 cells (**G**). Western blot analysis tested the related proteins levels using the indicated antibodies in HepG2 cells (**H**). Western blot analysis tested the related proteins levels using the indicated antibodies in mice liver tissues (**I**). For (**A**) one-way ANOVA was performed, followed by Tukey’s multiple comparison test. In (**C**, **E**-**I**), unpaired student’s t test was used for statistical analysis. Data are expressed as mean ± SD. ##*P* < 0.01, ###*P* < 0.001 (vs. NCD or Control group), *P < 0.05, **P < 0.01, ***P < 0.001 (vs. HFD or FFA group), n.s. not significant (*P* > 0.05)

Based on the in vivo experiments, we further established an in vitro model to investigate the protective mechanism of γ-GC against MASLD. HepG2 cells and primary hepatocytes were treated with increasing concentrations of γ-GC for 48 h to determine an appropriate working range. The CCK-8 assay showed that γ-GC (up to 320 µM) did not significantly alter the CCK-8 readout in HepG2 cells or primary hepatocytes (Fig. 3A), suggesting no apparent impairment of cellular metabolic activity under these conditions. To substantiate the absence of overt cytotoxicity beyond metabolic readouts, membrane integrity was further assessed by LDH release, which was not increased by γ-GC at the tested concentrations (Fig. 3B). Therefore, γ-GC with doses of 20 µM, 40 µM and 80 µM were selected for subsequent experiments. To establish an in vitro cell model of MASLD, we stimulated HepG2 cells and primary hepatocytes with 1mM FFA (OA/PA = 2/1) [34]. FFA treatment at the indicated concentration did not cause significant impairment of cellular metabolic activity, as assessed by CCK-8 assay (Fig. 3C). ORO staining was performed to verify the successful establishment of the cell model. As shown in Fig. 3D, both HepG2 cells and primary hepatocytes showed significant lipid droplets after FFA stimulation, whereas γ-GC treatment reduced the production of lipid droplets in a dose-dependent manner. Compared with FFA group, the intracellular levels of TG (Fig. 3E), TC (Fig. 3F) and FFAs (Fig. 3G) in HepG2 cells were also remarkably decreased by γ-GC treatment, which fully demonstrated the inhibition of γ-GC on FFA-induced lipid deposition. To further explore the mechanism by which γ-GC reduces lipid deposition, we examined several lipogenesis-related proteins. ACC1 and FASN are key lipogenic enzymes in the liver, while SREBP-1c is an important transcriptional regulator of lipogenic gene expression [35]. Immunoblotting results showed that FFA stimulation increased the protein levels of SREBP-1c, ACC1, and FASN, whereas γ-GC significantly reduced their expression (Fig. 3H). Since LXRα and PPARγ are also important regulators involved in lipid metabolic regulation, we further examined their protein levels. As expected, γ-GC treatment remarkably reduced the protein levels of LXRα and PPARγ (Fig. 3H), which was also confirmed in vivo (Fig. 3I). Overall, these results suggested that γ-GC attenuates FFA-associated lipogenic signaling and thereby contributes to the reduction of lipid deposition.

### γ-GC reduces fatty acid uptake by regulating the plasma membrane translocation of CD36

**Fig. 4:**
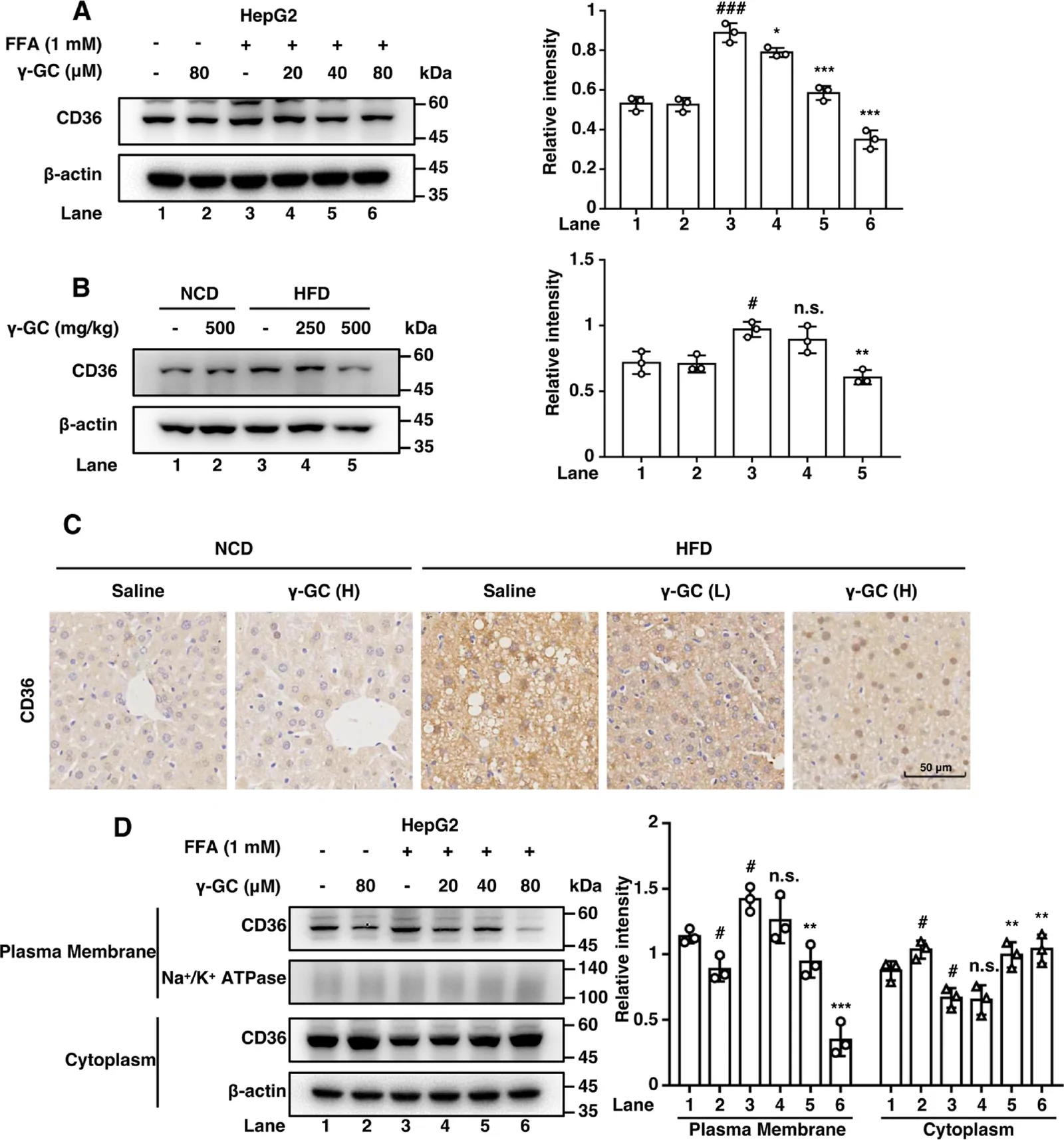
γ-GC reduces fatty acid uptake by regulating the intracellular localization of CD36.HepG2 cells were stimulated with 1 mM FFA for 24 h, then treated cells with different doses of γ-GC (0, 20, 40, 80 µM) for another 24 h. Western blot analysis tested the related proteins levels using the indicated antibodies in HepG2 cells (**A**, **D**). Western blot analysis tested the related proteins levels using the indicated antibodies in mice liver tissues (**B**). Representative images of immunohistochemical staining of CD36 in liver sections. Scale bar:50 μm. (**C**). In (**A**-**D**), unpaired student’s t test was used for statistical analysis. Data are expressed as mean ± SD. #*P* < 0.05, ###*P* < 0.001 (vs. NCD or Control group), *P < 0.05, **P < 0.01, ***P < 0.001 (vs. HFD or FFA group), n.s. not significant (*P* > 0.05)

The uptake of fatty acid from plasma in the liver is mainly dependent on the fatty acid transferase CD36 located on the cell surface [36]. In order to investigate the regulation of γ-GC on fatty acid uptake, we focused on the relationship between γ-GC and CD36 in the following experiments. Results showed that γ-GC stimulation inhibited the up-regulation of CD36 protein level induced by FFA stimulation to reduce hepatic lipid deposition (Fig. 4A, B). Additionally, the protein level of CD36 in the liver tissue by immunohistochemical experiment was consistent with the results from immunoblotting assay (Fig. 4C). Next, we performed the plasma membrane separation experiment in HepG2 cells, followed by immunoblotting assay, and it was found that the protein level of CD36 increased in the cell plasma membrane and decreased in the cytoplasm after FFA stimulation, indicating that FFA promoted the translocation of CD36 from cytoplasm to plasma membrane, which was reversed by γ-GC administration (Fig. 4D). The results indicated that γ-GC inhibits the plasma membrane translocation of CD36 to reduce fatty acid uptake.

### γ-GC inhibits the palmitoylation of CD36 by regulating the expression of zDHHC7

**Fig. 5:**
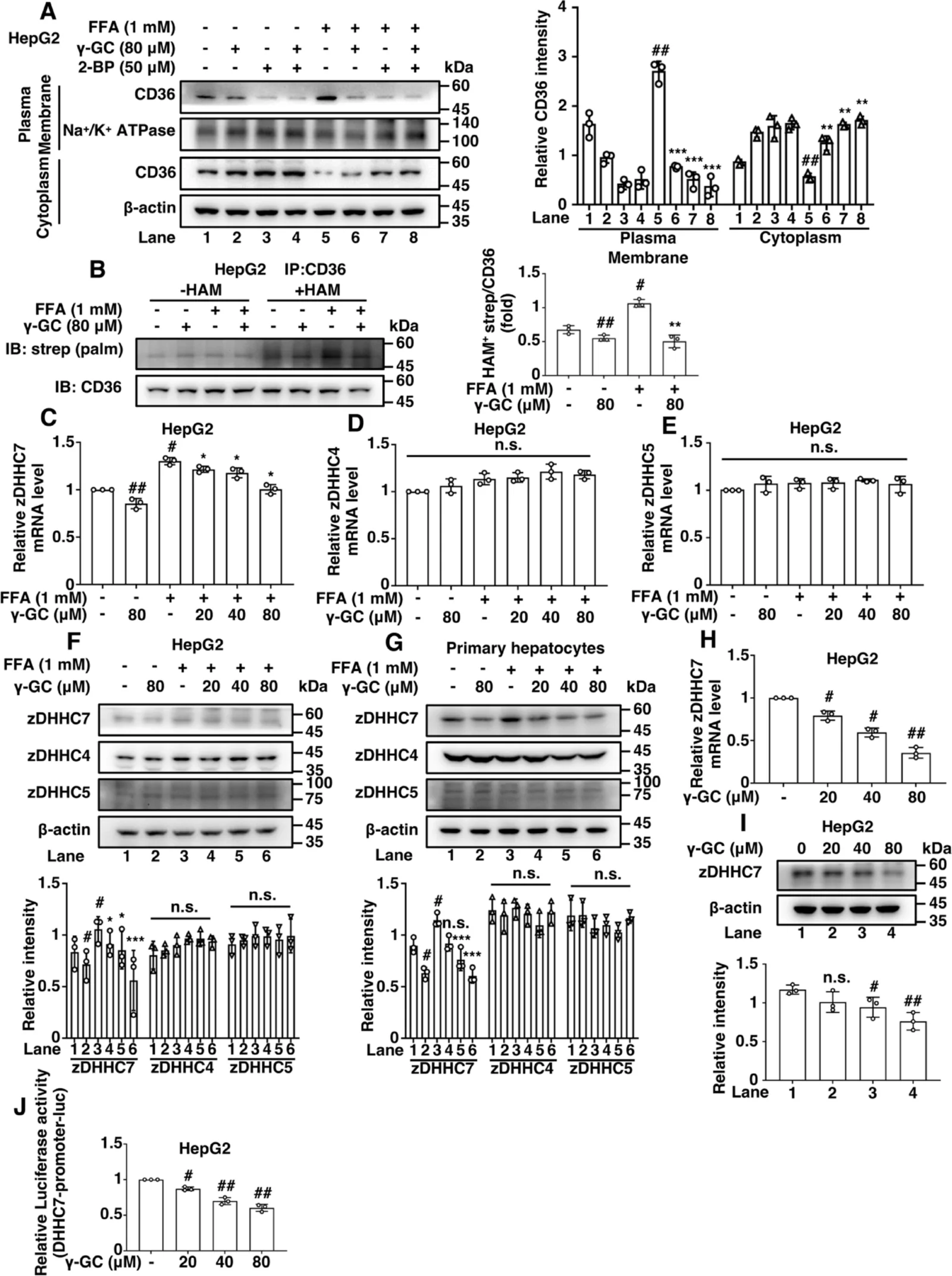
γGC inhibits the palmitoylation of CD36 by regulating the expression of zDHHC7.HepG2 cells were stimulated with 1 mM FFA for 24 h, then treated cells with 80 µM γ-GC or 50 µM 2-BP for another 24 h. Western blot analysis tested the related proteins levels using the indicated antibodies in HepG2 cells (**A**). HepG2 cells were stimulated with 1 mM FFA for 24 h, then treated cells with 80 µM γ-GC for another 24 h. IP-ABE assay and western blot analysis were used to evaluate the palmitoylation of CD36 (**B**). HepG2 cells were stimulated with 1 mM FFA for 24 h, then treated cells with different doses of γ-GC (0, 20, 40, 80 µM) for another 24 h. qPCR analyzed the levels of *Zdhhc7*, *Zdhhc4* and *Zdhhc5* mRNA in HepG2 cells (**C**, **D**, **E**). Western blot analysis tested the related proteins levels using the indicated antibodies in HepG2 cells (**F**) and primary hepatocytes (**G**). HepG2 cells were stimulated with different doses of γ-GC (0, 20, 40, 80 µM) for 24 h. qPCR analyzed the levels of *Zdhhc7* mRNA in HepG2 cells (**H**). Western blot analysis tested the related proteins levels using the indicated antibodies in HepG2 cells (**I**). Double luciferase reporter gene assay was used to detect the promoter activity of zDHHC7 in HepG2 cells (**J**). For (**D**, **E**) one-way ANOVA was performed, followed by Tukey’s multiple comparison test. In (**A**, **B**, **C**, **F**, **G**, **H**, **I**, **J**), unpaired student’s t test was used for statistical analysis. Data are expressed as mean ± SD. #*P* < 0.05, ##*P* < 0.01 (vs. Control group), *P < 0.05, **P < 0.01, ***P < 0.001 (vs. FFA group), n.s. not significant (*P* > 0.05)

Protein palmitoylation is one of the most common lipid modifications in which C16 fatty acid chains are covalently linked to cysteine residues via a thioester linkage [37]. It has been reported that the palmitoylation modification of CD36 promotes its translocation into plasma membrane. Since our above results have demonstrated that γ-GC has a regulatory effect on the plasma membrane translocation of CD36, we next used 2-BP (an inhibitor of palmitoylation modification) to investigate whether γ-GC regulates palmitoylation modification of CD36. When HepG2 cells were treated with FFA, both γ-GC and 2-BP showed the same inhibitory effect on the translocation of CD36 to plasma membrane (Fig. 5A). Then, we performed IP-ABE experiment to detect the palmitoylation of CD36. As shown in Fig. 5B, γ-GC could significantly inhibit CD36 palmitoylation induced by FFA in HepG2 cells, which preliminarily proved that γ-GC could inhibit the palmitoylation of CD36. Although the modification of S-acylation/palmitoylation was first discovered in the 1970s [38], a large family of DHHC-containing PATs that catalyze the reaction were identified only in early 2000s [39, 40]. More than 20 PATs have been identified in mammals, of which zDHHC4, zDHHC5 and zDHHC7 have been shown to be key PATs in regulating the palmitoylation of CD36 [41, 42]. Using qPCR and immunoblotting assay to detect whether γ-GC has the regulatory effect on zDHHC4, zDHHC5 and zDHHC7. Results showed that γ-GC specifically targeted zDHHC7 instead of zDHHC4 and zDHHC5 both in HepG2 cells and in primary hepatocytes (Fig. 5C-G). As Fig. 5H and I showed, γ-GC inhibited the mRNA and protein level of zDHHC7. Further research confirmed that γ-GC could inhibit the transcription of zDHHC7, which was verified by using the luciferase reporter gene with zDHHC7 promoter region to perform the double luciferase reporter gene assay (Fig. 5J). These above results demonstrated that γ-GC inhibits CD36 palmitoylation by impeding the transcriptional activity of zDHHC7 promoter.

### γ-GC inhibits CD36 plasma membrane translocation via modulating AKT/KLF10/zDHHC7 signaling

**Fig. 6:**
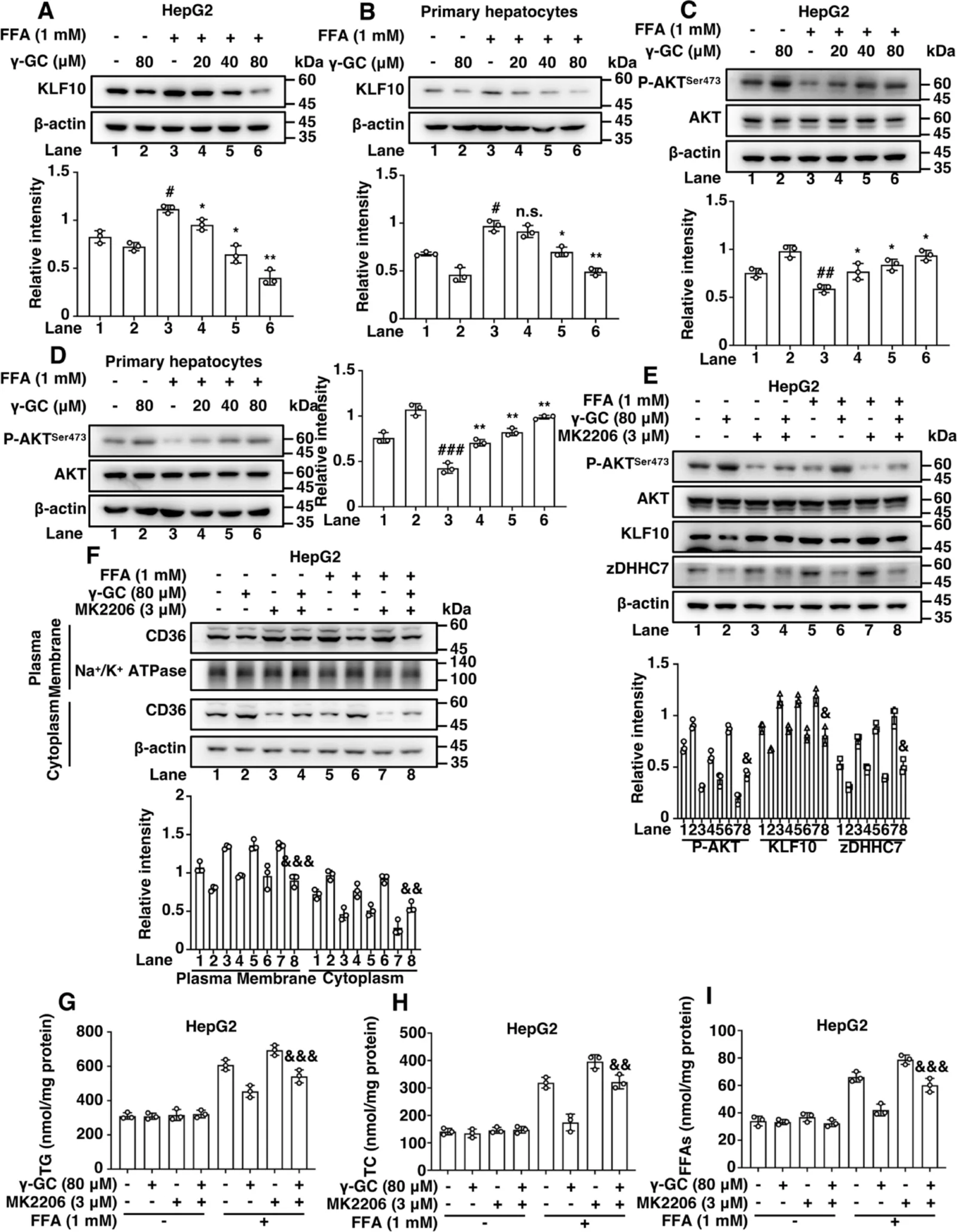
γGC inhibits CD36 plasma membrane translocation via modulating AKT/KLF10/zDHHC7 signaling.HepG2 cells or primary hepatocytes were stimulated with 1 mM FFA for 24 h, then treated cells with different doses of γ-GC (0, 20, 40, 80 µM) for another 24 h. Western blot analysis tested the related proteins levels using the indicated antibodies (**A**-**D**). HepG2 cells were stimulated with 1 mM FFA for 24 h, then treated cells with 80 µM γ-GC or 3 µM MK2206 for another 24 h. Western blot analysis tested the related proteins levels using the indicated antibodies in HepG2 cells (**E**, **F**). Intracellular TG, TC, and FFAs levels were measured by commercial kits (**G**-**I**). In (**A**-**I**), unpaired student’s t test was used for statistical analysis. Data are expressed as mean ± SD. #*P* < 0.05, ##*P* < 0.01, ###*P* < 0.001 (vs. Control group), *P < 0.05, **P < 0.01 (vs. FFA group), &*P* < 0.05, &&*P* < 0.01, &&&*P* < 0.001 (vs. FFA + MK2206 group), n.s. not significant (*P* > 0.05)

KLF10, also known as TGF-β-inducible early gene 1 (TIEG1), is a key regulator of cellular homeostasis and plays a role in growth, development and programmed cell death. Studies have shown that KLF10, as the upstream transcription factor of zDHHC7, can regulate the expression of zDHHC7, and then participate in the liver glucose and lipid homeostasis [42]. In order to clarify the molecular mechanism of γ-GC inhibiting the palmitoylation of CD36 via regulating zDHHC7, we further examined the protein level of KLF10. When FFA stimulated cells, the protein level of KLF10 increased significantly; however, γ-GC administration could reduce the protein level of KLF10 dose-dependently (Fig. 6A, B). As AKT has been reported to bridge γ-GC and the plasma membrane localization of CD36 through its phosphorylation [22], we speculated that AKT, as the upstream of KLF10, participates in the process of which γ-GC regulates the plasma membrane localization of CD36. As expected, γ-GC administration inhibited the FFA-induced reduction in the phosphorylation of AKT (Fig. 6C, D). Because AKT phosphorylation was assessed under FFA-loading conditions without acute insulin stimulation, these data should be interpreted as reflecting modulation of AKT signaling rather than a direct assessment of insulin responsiveness. These results provided preliminary evidences that γ-GC could inhibit the KLF10/zDHHC7-induced plasma membrane localization of CD36 by activating AKT. To interrogate the involvement of AKT signaling, the AKT inhibitor MK2206 was applied in subsequent experiments. MK2206 markedly reduced AKT phosphorylation and was accompanied by increased protein levels of KLF10 and zDHHC7. Notably, co-treatment with γ-GC significantly blunted the MK2206-induced upregulation of KLF10 and zDHHC7 (Fig. 6E). We next examined CD36 subcellular localization and found that MK2206 promoted CD36 translocation to the plasma membrane, whereas γ-GC co-treatment significantly attenuated this effect (Fig. 6F). Consistently, biochemical analyses showed that MK2206 exacerbated FFA-induced lipid accumulation, as evidenced by elevated intracellular TG, TC, and FFAs in HepG2 cells; γ-GC significantly mitigated these increases (Fig. 6G-I). Collectively, these data support the involvement of AKT signaling in γ-GC-mediated regulation of the KLF10/zDHHC7 signaling, thereby influencing CD36 trafficking and lipid deposition.

We next examined whether CD36, KLF10, and ZDHHC7 were dysregulated in human steatosis/MASH liver using two independent GEO cohorts (Fig. S1A; Table S1, 2). In GSE89632, CD36 showed no significant change in Steatosis vs. Control but was significantly upregulated in MASH vs. Control. In contrast, KLF10 was significantly downregulated in both Steatosis vs. Control and MASH vs. Control. ZDHHC7 did not show significant differences in Steatosis vs. Control or MASH vs. Control. In GSE48452, CD36 displayed an upward trend in Steatosis vs. Control but did not remain significant after multiple-testing correction, and was not significant in MASH vs. Control. KLF10 and ZDHHC7 were not significantly altered in Steatosis vs. Control or MASH vs. Control. Overall, across two independent human liver cohorts, CD36 upregulation is supported most robustly in the MASH group of GSE89632 and shows a consistent directionality in GSE48452, whereas KLF10 downregulation is strongly supported in GSE89632 but not replicated in GSE48452. ZDHHC7 mRNA abundance did not show significant alterations in bulk liver transcriptome profiles in either cohort, which may reflect cohort heterogeneity and the limitation that palmitoylation-related regulation may not be fully captured at the transcript level in bulk tissue. We next evaluated whether these genes exhibit coordinated expression patterns within the same histologic stage using Spearman correlation. Within steatosis, pairwise correlations were generally weak and non-significant in both cohorts; notably, ZDHHC7-CD36 showed a trend-level positive association in GSE89632, which was not recapitulated in GSE48452 (Fig. S1B). Within MASH, most correlations were likewise non-significant; KLF10-CD36 showed a trend toward positive association in GSE48452, whereas the corresponding association in GSE89632 was in the opposite direction and not significant (Fig. S1C). Together, these human transcriptomic analyses indicate that selected components of lipid metabolic and palmitoylation-related pathways are altered in steatotic/MASH liver. However, because KLF10 was downregulated in these datasets, the human data provide only partial and mixed support for the proposed KLF10/zDHHC7/CD36 palmitoylation pathway.

**Fig. 7:**
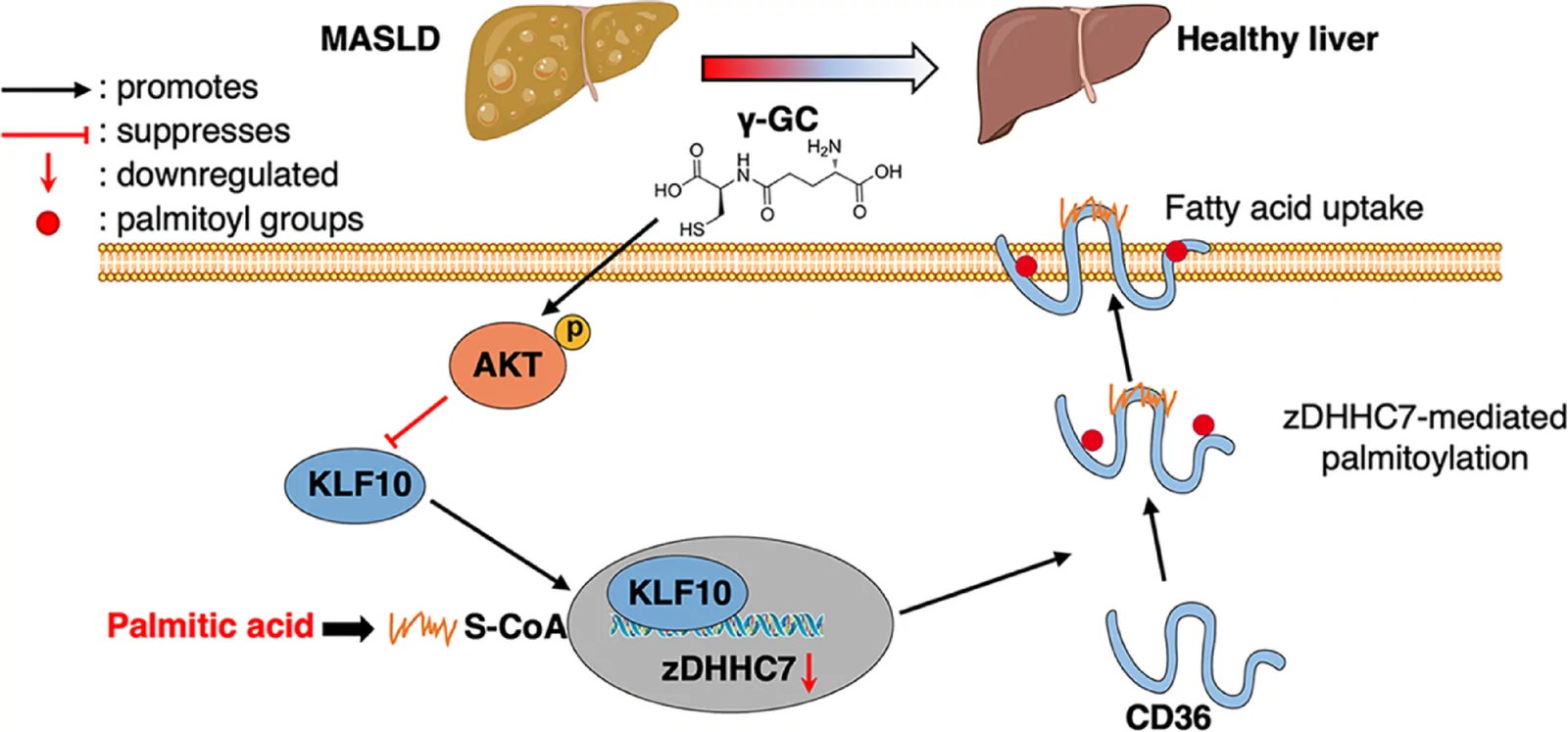
γ-GC mitigates MASLD by inhibiting KLF10/zDHHC7-mediated CD36 palmitoylation and plasma membrane translocation via activating AKT

## Discussion

MASLD is the most prevalent chronic liver condition, impacting approximately 32.4% of the world’s population [43]. Studies have shown that the clinical burden of MASLD is not limited to liver-related morbidity and mortality, but also includes serious damage to extrahepatic organs and regulatory pathways. As a result, MASLD increases the risk of cardiovascular diseases, atherosclerosis, diabetes and central nervous system diseases [44]. The pathological spectrum of MASLD includes isolated fatty liver (simple steatosis) and metabolic dysfunction-related steatohepatitis (MASH), the inflammatory sub phenotype of MASLD that can lead to progressive liver fibrosis, cirrhosis and hepatocellular carcinoma (HCC) [45]. Although resmetirom, an agonist of thyroid hormone receptor β, has been shown to be useful for the treatment of MASH, no other specific drugs for treating MASLD have been discovered yet [46]. It is critically important to develop additional novel therapeutics that can effectively ameliorate MASLD, thereby offering patients a wider array of choices for management. Our latest study has reported that γ-GC alleviates IR in diabetic mice and reduces lipid ectopic deposition in the liver, suggesting that γ-GC may have a protective effect on MASLD [22].

Prolonged consumption of an exogenous HFD and caloric excess are key contributors to the progression of MASLD [47]. Consequently, we fed mice with HFD (60 kcal% fat diet) for 16 weeks during in vivo experiments [24]. Meanwhile, we conducted in vitro experiments using FFA to induce lipid accumulation in HepG2 cells and primary hepatocytes. In the current study, γ-GC treatment was associated with improved fasting glycemic indices, including reduced fasting glucose and insulin levels and a lower HOMA-IR index, supporting an attenuation of systemic insulin resistance under fasting conditions in HFD-fed mice. A change in body weight was associated with the remission of MASLD [48]. γ-GC treatment reduced both body weight and adipose tissue mass in HFD-fed mice, which may contribute to the remission of MASLD. In the present study, we also observed a significant reduction in food intake in the γ-GC treated group compared to the HFD group. Given that caloric restriction is well-documented to alleviate dyslipidemia and metabolic syndrome, this reduced food intake likely contributes partially to the observed improvements in the lipid phenotype. Nevertheless, our in vitro data and the modulation of specific molecular markers strongly suggest that γ-GC also exerts direct regulatory effects on hepatic lipid metabolism. The improved hepatic lipid phenotype in vivo may reflect both reduced food intake and direct metabolic actions of γ-GC; however, in the absence of a pair-feeding design, their relative contributions cannot be definitively separated. Since the liver is a crucial organ for glucose and lipid metabolism, lipid ectopic deposition in the liver is a significant factor leading to the development of MASLD. Dyslipidemia is a clinical characteristic of MASLD, traditionally assessed based on levels of TG, TC, HDL-C, and LDL-C [1]. ALT and AST, being critical indicators for assessing liver function, are associated with blood lipid levels. Therefore, the amelioration of dyslipidemia can mitigate liver damage. In this study, γ-GC not only reversed dyslipidemia through reducing serum levels of TG, TC and LDL while increasing HDL levels, but also ameliorated liver dysfunction by decreasing serum ALT and AST levels. Furthermore, γ-GC also ameliorated hepatic steatosis and reduced liver coefficient associated with liver hypertrophy caused by HFD. γ-GC, as an immediate precursor of glutathione, is expected to support GSH synthesis. Our data showing an increased hepatic GSH/GSSG ratio together with decreased lipid peroxidation markers suggest that γ-GC restores redox homeostasis in MASLD liver. Given that oxidative stress can exacerbate lipotoxicity, inflammation, and insulin resistance, redox restoration may represent an important mechanism linking γ-GC to improved hepatic metabolic phenotypes.

An imbalance between lipid acquisition and lipid disposal results in hepatic lipid accumulation, which is regulated by fatty acid uptake, DNL, fatty acid oxidation (FAO), and the export of lipids in very low-density lipoproteins (VLDL) [49]. In brief, DNL allows the liver to synthesize new fatty acids from acetyl-CoA. Acetyl-CoA is first converted into malonyl-CoA by ACC1, which is subsequently used by FASN for fatty acid synthesis. SREBP-1c, a key transcriptional regulator of hepatic lipogenic gene expression, is regulated by LXRα and can activate the lipogenic pathway [50]. Consequently, elevated DNL can lead to hepatic steatosis and hypertriglyceridemia. Additionally, it may contribute to steatohepatitis, as saturated fatty acids such as palmitate have the potential to trigger inflammation and apoptosis [51]. During our experiments, γ-GC attenuated the FFA-associated lipogenic program, as reflected by reduced expression of SREBP-1c, ACC1, FASN, LXRα, and PPARγ, thereby contributing to the alleviation of hepatic lipid accumulation. γ-GC has been reported to alleviate ethanol-induced hepatotoxicity via suppressing oxidative stress, apoptosis, and inflammation [23]; however, we could not judge whether γ-GC can inhibit steatohepatitis induced by lipid accumulation in the current experiment. In the future, more experiments are needed to investigate the relationship between γ-GC and steatohepatitis. The uptake of circulating fatty acids by the liver primarily relies on fatty acid transporters [52]. CD36, a fatty acid transporter widely expressed in various cell types, is mainly responsible for fatty acid uptake. Studies have shown that CD36 can also promote DNL in hepatocytes through INSIG2-dependent SREBP1 processing [9]. Elevated expression of CD36 in the liver is strongly linked to IR, hyperinsulinemia, and steatosis in individuals suffering from MASH [13]. Therefore, CD36, as a key factor in regulating lipid metabolism, can induce lipid accumulation by increasing lipid acquisition. Here, γ-GC has been proved to inhibit hepatic lipid accumulation through reducing the protein level of CD36 both in vivo and in vitro.

In patients with steatosis and MASH, CD36 is located in the plasma membrane of hepatocyte, whereas in normal liver, weak expression of CD36 is confined to the cytoplasm of hepatocyte, suggesting that translocation of CD36 from cytoplasm to plasma membrane may trigger the progression of MASLD [53]. Palmitoylation of CD36 is required for its plasma membrane localization and fatty acid uptake [12]. Interestingly, our results demonstrated that γ-GC can not only reduce CD36 protein level, but also decrease fatty acid uptake by regulating CD36 intracellular localization. Using the palmitoylation inhibitor 2-BP, it was further confirmed that γ-GC can block the targeting of CD36 to the plasma membrane by inhibiting CD36 palmitoylation. Our current data do not allow us to conclude that the effect of γ-GC on lipogenesis-related signaling depends on the regulation of CD36 palmitoylation and plasma membrane translocation, and this will need to be further clarified in future studies.

S-palmitoylation is catalyzed by PATs, also named zinc finger DHHC-type containing (zDHHC) proteins, and dysregulation of palmitoylation is associated with various diseases, such as infections, cancer, metabolic syndrome and neurological disorders [54]. As zDHHC4, zDHHC5 and zDHHC7 have been reported to facilitate fatty acid uptake by palmitoylating and targeting CD36 to the plasma membrane [41, 55], we explored the effect of γ-GC on the expressions of zDHHC4, zDHHC5 and zDHHC7. Interestingly, only zDHHC7 expression was effectively regulated by γ-GC, and γ-GC could significantly inhibit the transcriptional activity of zDHHC7 promoter. KLF10, a key regulator of intracellular homeostasis, regulates the transcriptional activity and expression of zDHHC7 [42]. As expected, γ-GC was verified to inhibit KLF10-regulated zDHHC7 transcription by reducing the protein level of KLF10. In previous study, we have demonstrated that γ-GC inhibits the translocation of CD36 to the plasma membrane via activating AKT signaling, but the specific molecular mechanism remains unclear. The expression of KLF15 can be negatively regulated by PI3K/AKT signaling [56]; however, few studies have been reported on the regulation of KLF10 expression by AKT signaling. Currently, we verified that γ-GC activated FFA-suppressed AKT signaling both in HepG2 cells and primary hepatocytes. Because acute insulin stimulation was not applied in these experiments, the observed changes in AKT phosphorylation should be interpreted as alterations in AKT signaling under FFA-loading conditions rather than a direct measurement of insulin-stimulated insulin responsiveness. Furthermore, inhibition of phosphorated AKT by MK2206 promoted KLF10/zDHHC7-mediated CD36 palmitoylation and plasma membrane translocation, ultimately leading to lipid accumulation in hepatic. However, γ-GC administration significantly reversed these phenomena. The stability of KLF10 was shown to be mediated by FBW7 [57]. Whether AKT regulates KLF10 protein level through FBW7 remains unclear and needs to be further verified in future experiments. The human transcriptomic findings should be interpreted with caution. Although they suggest possible relevance of selected downstream components of the pathway, KLF10 was downregulated in steatotic/MASH liver, which is not fully consistent with our experimental model. This discrepancy may reflect the complexity and heterogeneity of human MASH, differences between bulk transcriptomic datasets and hepatocyte-specific regulatory events, disease-stage-dependent regulation of KLF10, or discordance between mRNA levels and protein activity. Therefore, the human datasets do not provide direct validation of the proposed mechanism, and further studies using cell-type-specific and protein-level analyses in human liver samples are required.

Building on prior work describing the KLF10/zDHHC7/CD36 axis in hepatic lipid metabolism [42], our study suggests that γ-GC functions as an upstream modulator of this previously reported pathway. We found that γ-GC attenuated hepatic lipid accumulation by reducing fatty acid uptake and suppressing lipogenesis-related signaling, thereby alleviating MASLD-related phenotypes. Mechanistically, γ-GC suppressed KLF10/zDHHC7-dependent CD36 palmitoylation and reduced CD36 plasma membrane localization. Notably, γ-GC increased AKT phosphorylation, and pharmacological inhibition of AKT attenuated the γ-GC-induced suppression of the KLF10/zDHHC7/CD36 axis, supporting a role for AKT signaling in transmitting γ-GC-dependent regulatory cues to this axis. Future studies are warranted to determine whether γ-GC also impacts liver fibrosis and to benchmark its efficacy against resmetirom, which is approved for noncirrhotic MASH/NASH with moderate-to-advanced fibrosis.

## Conclusion

In conclusion, our findings indicate that γ-GC mitigates hepatic lipid accumulation by limiting fatty acid uptake and attenuating lipogenesis-related signaling. Moreover, this study adds a new regulatory layer by identifying γ-GC as a potential upstream modulator of the established KLF10/zDHHC7/CD36 pathway, with pharmacological evidence supporting the involvement of AKT signaling in this regulation (Fig. 7). These results provide a rationale for further preclinical evaluation of γ-GC as a therapeutic lead or adjunct strategy for MASLD.

## Supplementary Information

Below is the link to the electronic supplementary material.

Supplementary Material 1.

## Data Availability

The data that support the findings of the present study are available from the corresponding author on reasonable request. The public datasets analyzed during the current study are available in the Gene Expression Omnibus repository, and the URL: https://identifiers.org/geo: GSE48452, https://identifiers.org/geo: GSE89632.

## Abbreviations

ALD: Alcoholic liver disease
FAO: Fatty acid oxidation
FFAs: Free fatty acids
GSH: Glutathione
GSS: Glutathione synthetase
HCC: Hepatocellular carcinoma
HDL-C: High-density lipoprotein cholesterol
HFD: High-fat diet
IR: Insulin resistance
LDL-C: Low density lipoprotein cholesterol
MASH: Metabolic dysfunction-related steatohepatitis
MASLD: Metabolic dysfunction–associated steatotic liver disease
NAFLD: Nonalcoholic fatty liver disease
OA: Oleic acid
ORO: Oil red o
PA: Palmitic acid
PATs: Palmitoyl acyl transferases
PFA: Paraformaldehyde
SAT: Subcutaneous adipose tissue
TC: Total cholesterol
TG: Triglyceride
TIEG1: TGF-β-inducible early gene 1
VAT: Visceral adipose tissue
VLDL: Very low-density lipoproteins
γ-GC: γ-glutamylcysteine
