# Supplementary material for: γ-GC mitigates MASLD by reducing CD36 palmitoylation and plasma membrane translocation mediated by AKT/KLF10/zDHHC7 axis
Source: Nutr Metab (Lond). 2026 Jul 12;23:118. doi: 10.1186/s12986-026-01173-6 (PMC13647763; doi:10.1186/s12986-026-01173-6)
Supplement: Supplementary file 1 [file 12986_2026_1173_MOESM1_ESM.docx]

**Supplementary materials**

**Fig.S1** **Public human liver transcriptomes evaluate differential expression and co-variation of CD36, KLF10, and ZDHHC7 across Steatosis/MASH stages**

Box-and-jitter plots of processed expression values for Control, Steatosis, and MASH (**A**). Spearman co-variation among KLF10, ZDHHC7, and CD36 within Steatosis samples (**B**). Spearman co-variation among KLF10, ZDHHC7, and CD36 within MASH samples (**C**).

| gene (GSE89632) | contrast | delta_expression | t | *P* | n | FDR_BH |
| --- | --- | --- | --- | --- | --- | --- |
| CD36 | Steatosis-Control | 0.069041229 | 0.838376717 | 0.405146754 | 63 | 0.405146754 |
| CD36 | NASH-Control | 0.210527249 | 2.520536105 | 0.014394809 | 63 | 0.021592214 |
| CD36 | NASH-Steatosis | 0.14148602 | 1.623713636 | 0.109679657 | 63 | 0.292593884 |
| KLF10 | Steatosis-Control | -0.788167417 | -4.591802659 | 0.000023005 | 63 | 0.000069015 |
| KLF10 | NASH-Control | -0.554405654 | -3.184537018 | 0.002299969 | 63 | 0.006899907 |
| KLF10 | NASH-Steatosis | 0.233761763 | 1.287073732 | 0.203011834 | 63 | 0.292593884 |
| ZDHHC7 | Steatosis-Control | -0.125956175 | -1.425317255 | 0.159247119 | 63 | 0.238870678 |
| ZDHHC7 | NASH-Control | -0.026673289 | -0.297592903 | 0.767041876 | 63 | 0.767041876 |
| ZDHHC7 | NASH-Steatosis | 0.099282886 | 1.061773017 | 0.292593884 | 63 | 0.292593884 |

**Table.S1 Differential expression and co-variation analyses of CD36, KLF10, and ZDHHC7 in public human liver cohorts (GSE89632).**

| gene (GSE48452) | contrast | delta_expression | t | *P* | n | FDR_BH |
| --- | --- | --- | --- | --- | --- | --- |
| CD36 | Steatosis-Control | 0.377149505 | 2.073347051 | 0.044165224 | 46 | 0.132495673 |
| CD36 | NASH-Control | 0.261862103 | 1.526888678 | 0.134111572 | 46 | 0.402334716 |
| CD36 | NASH-Steatosis | -0.115287401 | -0.672227961 | 0.505034824 | 46 | 0.613427721 |
| KLF10 | Steatosis-Control | -0.25447187 | -1.40794931 | 0.166334988 | 46 | 0.249502482 |
| KLF10 | NASH-Control | -0.024038795 | -0.141070495 | 0.888473072 | 46 | 0.888473072 |
| KLF10 | NASH-Steatosis | 0.230433075 | 1.352285262 | 0.183353584 | 46 | 0.550060753 |
| ZDHHC7 | Steatosis-Control | 0.101185643 | 1.129901009 | 0.264781517 | 46 | 0.264781517 |
| ZDHHC7 | NASH-Control | 0.058219155 | 0.689546655 | 0.4941839 | 46 | 0.741275849 |
| ZDHHC7 | NASH-Steatosis | -0.042966488 | -0.508894343 | 0.613427721 | 46 | 0.613427721 |

**Table.S2 Differential expression and co-variation analyses of CD36, KLF10, and ZDHHC7 in public human liver cohorts (GSE48452).**
